# Is cyberbullying perpetration associated with anxiety, depression and suicidal ideation among lebanese adolescents? Results from a cross-sectional study

**DOI:** 10.1186/s40359-023-01091-9

**Published:** 2023-02-24

**Authors:** Zeinab Bitar, Marie-Belle Elias, Diana Malaeb, Souheil Hallit, Sahar Obeid

**Affiliations:** 1 Faculty of medicine, Paris-Saclay University, Le Kremlin-Bicêtre, France; 2 School of Arts and Sciences, Holy Spirit University of Kaslik, P.O. Box 446, Jounieh, Lebanon; 3 College of Pharmacy, Gulf Medical University, P.O. Box 4184, Ajman, United Arab Emirates; 4 School of Pharmacy, Lebanese International University, Beirut, Lebanon; 5 School of Medicine and Medical Sciences, Holy Spirit University of Kaslik, P.O. Box 446, Jounieh, Lebanon; 6 Applied Science Research Center, Applied Science Private University, Amman, Jordan; 7 Research Department, Psychiatric Hospital of the Cross, Jal Eddib, Lebanon; 8 Social and Education Sciences Department, School of Arts and Sciences, Lebanese American University, Jbeil, Lebanon

**Keywords:** Cyberbullying perpetration, Adolescents, Anxiety, Depression, Household Crowding Index, Suicidal ideation, Lebanon

## Abstract

**Background:**

As cyberbullying is a new area of investigation, results worldwide point to the prevalence of cyberbullying perpetration. This study aimed to assess the association between cyberbullying perpetration, anxiety, depression and suicidal ideation among Lebanese adolescents.

**Methods:**

This cross-sectional study was conducted between May and June 2021 and included a sample of adolescents aged between 13 and 16 years old, recruited from private schools chosen in a convenient way from all Lebanese districts. A total of 520 students accepted to participate in our study. To collect data, a questionnaire was shared by google form including: Cyber Bully/Cyber victim questionnaire; Lebanese Anxiety Scale; and Patient Health Questionnaire for Adolescents.

**Results:**

The results of the linear regressions, taking anxiety and depression as dependent variables, showed that female gender, having kind of hard and very/extremely hard influence of problems on daily work, sexual cyberbullying in cyberspace, embarrassing and inserting malicious content in cyberspace and older age were significantly associated with more anxiety and depression. Having kind of hard influence of problems on daily work compared to not at all, higher anxiety, higher depression and higher household crowding index (lower socioeconomic status) were significantly associated with higher odds of having suicidal ideation in the last month.

**Conclusion:**

Cyberbullying perpetration and its associated factors reported in this study are significant enough to call for early detection and prevention strategies for Lebanese adolescents. At the school level, effective programs implemented in the school years are needed, aiming to develop social/emotional control, and conflict resolution skills as they might decrease engagement in cyberbullying perpetration among adolescents. Preventive interventions are needed to reduce the engagement of Lebanese adolescents in cyberbullying perpetration.

## Background

Bullying represents one of the most serious public health issues. It is defined as a continual aggressive act committed by a group or an individual over time against a victim who is unable to defend him/herself [1]. Given the rapid development and the emergence of communication and information technology (i.e. text messages, smartphones, and social media platforms), new ways to take out frustrations and aggression among adolescents emerged. Adolescents can easily form a large number of online groups, which are difficult to control, with a potentially increased risk of becoming cyberbullies [2]. Hence, the term “Cyberbullying” referring to bullying through the internet has emerged and grabbed the attention of researchers and communities globally [3].

As cyberbullying is a new area of investigation [4], results worldwide point to the prevalence of cyberbullying perpetration [5]. The Middle East and North Africa region was named among the countries with the highest rates of bullying among adolescents aged 11 to 15 in 2018 [6]. In Lebanon, approximately 90% of bullying acts occur in schools, with 41.9% of adolescents being bullying perpetrators [7].

Cyberbullying is characterized by the absence of physical interaction; the victim has no opportunity to defend him/herself at all times; the chances of identifying and punishing perpetrators are pretty low [8]. Because cyberbullying perpetrators are usually anonymous, their harmful behavior might be exacerbated. Victims can be abused in the security of their own home, at any time, and even if the victim removed himself from the online site, the messages can still accumulate without being able to escape from this negative situation [9]. Unlike direct traditional bullying, which can be identified right away by parents or teachers, cyberbullying takes more time to be identified [9].

Excessive risky online use, moral disengagement, narcissism, self-efficacy, social norms, and substance use have all been identified as risk factors that increase a person’s likelihood of becoming a cyberbullying perpetrator [10]. Similarly, the outcomes of cyberbullying perpetration were found to be associated with a variety of problems such as depression, anxiety, loneliness, low self-esteem, aggressive cognition, moral disengagement, and substance use. It was also associated with lower academic achievement and life satisfaction [9, 11]. Furthermore, cyberbullies were at a higher risk of self-harm, suicidal ideation, and suicide attempts [12, 13].

The COVID-19 pandemic affected all people worldide and imposed strict restrictions such as social distancing, school closure, distanced learning experience, and online conferences, all of which increasing internet usage [14, 15]. In line with this situation, Lebanese were at a higher risk of social isolation, depression, loneliness, excessive internet use and cyberbullying, with 49% of adolescents being cyberbullying perpetrators [7, 16, 17]. Although multiple studies have addressed factors associated with cyberbullying perpetration occurrence, research thoroughly investigating consequences of this behavior is still needed to preempt incidents. Given the high rates of mental health issues among Lebanese adolescents (28.9% suicidal ideation [18] and 57.2% of moderate to high depressive symptoms [19]) and the rising prevalence of cyberbullying perpetration found among Lebanese adolescents and the crisis imposing higher internet use, we realized that it is important to shed the light on cyberbullying perpetration behavior and its consequences on bullies. Therefore, this study aimed to assess the association between cyberbullying perpetration and anxiety, depression and suicidal ideation among a sample of Lebanese adolescents.

## Methods

### Study design

This cross-sectional study was conducted between May and June 2021 and included a sample of adolescents aged between 13 and 16 years old, recruited from private schools chosen in a convenient way from all Lebanese districts. Following the restrictions imposed by the Lebanese government due to COVID-19 and the online teaching adopted by schools, the collection of the data was done using an online form. The questionnaire was tested on 25 students, prior to data collection, with the aim of clarifying questions if necessary, correcting errors and assessing the length of the questionnaire; the results of the pilot study was not included in the analysis. We contacted the directors of fifteen selected schools, explained the objective of the study and presented the questionnaire to them; the anonymity of the school and students was ensured. We contacted three schools in each governorate; the description of the data collection is summarized in Table 1.

**Table 1:** Description of data collection

District	Number of schools who agreed to pass the questionnaire to their students out of 3 schools approached in each district	Refusal reason(s)
North Lebanon	Two	Parents’ committee refused due to issues that address suicide
Mount Lebanon	Three	-
Beirut	Three	-
Bekaa	One	Questions related first to sexuality and then to suicide
South Lebanon	Two but with conditions: - Randomly choosing a class of each level (grades 7, 8 and 9). - Pass the questionnaire on the WhatsApp groups of the parents of the students of the chosen class to ensure the agreement of the parents by clicking on *‘I accept that my child participates in this study’* since the participants are minors.	Lack of personal conviction in the work in psychology

### Participants

The study sample consisted of 520 students from grades 7, 8 and 9 in private schools in all Lebanese districts; the governorates were classified according to their geographical proximity: Beirut, Mount Lebanon, North Lebanon (including Akkar), South Lebanon (including Nabatieh) and Bekaa (including Baalback-Hermel). All students randomly selected from classes and aged exactly 13 to 16 years of age (date of taking the tests) were eligible to take part of this study. Exclusions consisted of those who did not meet those criteria or refused to participate in this study. It should be noted that the objective of the study, the anonymity of the participants, the lack of personal profit, the agreement of the ethics committee, the lack of compensation of any kind, the freedom of choice to participate or not, were explained in the introductory paragraph of the Google Form. After approving those statements and asking for the parents’ approval, the student was guided to the actual questions.

### Minimal sample size calculation

According to the G-power software, and based on an effect size f2 = 2%, an alpha error of 5% and a power of 80%, while taking into account 20 factors to be captured in the multivariate analysis, the results showed that a minimum number of 395 participants was needed to conduct this study.

### Questionnaire

The questionnaire consisted of three measuring instruments used to separately assess each of the four variables studied and questions studying the socio-demographic characteristics of the participants (age, gender, parents’ status, influence of problems on work (including all student behaviors at school and associated consequences such as school dropout, getting remarks and repeating a year), and household crowding index). The latter reflects the socio-economic status of the family and was calculated by dividing the number of people by the number of rooms in the house, except the bathrooms and the kitchen [20]. In addition, a question about the presence of suicidal ideation the last month has been asked to students with a dichotomous answer (yes/no). The digital questionnaire was self-administered in Arabic, the participants’ mother tongue. The calculated time to complete the questionnaire was fifteen minutes.

#### Cyber bully/cyber victim questionnaire

The “Cyber Bully/Cyber Victim Scale” has been translated into Arabic via the ‘Forward-and-Backward’ method. This scale was translated first from English to Arabic by a professional translator, secondly from Arabic to English by another professional translator. A comparison was made between the original English version and the English translated copy by a mental health professional, with no major corrections made to the final Arabic version. The Cyber Bully/Cyber Victim Questionnaire was used to assess cyberbullying behavior [21]. Authorization to translate and use the scale was obtained from Dr. Mehmet Horzum. This scale consists of 15 items and is divided into two sub-dimensions: the first is composed of seven items and evaluates cyber sexual harassment in cyberspace, and the second is composed of eight items and evaluates the insertion of embarrassing and malicious content on cyberspace. It should be noted that this scale is used to assess at the same time or separately the behavior of perpetration and/or victimization. We used only the part about the harasser. Questions were rated from 1 = Never to 5 = all the time. Points are scored by adding all the points of the elements on the scale. The total score on “made by me” is considered the cyber stalker’s score and the total score on “made for me” is considered the cyber-victim score. In our study, a higher score on “made by me” items indicates a higher level of harassing behavior.

#### Lebanese anxiety scale (LAS-10)

This ten-item self-report scale was developed in Lebanon and validated among adults [22], and adolescents [23]. Seven of the ten items are rated on a 5-point Likert scale, whereas the other three questions are scored on a 4-point Likert scale [22]. The total score was obtained by adding up all responses, with higher scores indicating higher anxiety.

#### Patient health questionnaire for adolescents (PHQ-A)

This scale was being tested for use in adolescents and provides an effective tool for the early detection and recognition of mental disorders in this high-risk group [24]. The Arabic translation was validated by Al-Amer et al. (2020) [25]. It is a scale composed of (1) nine items that can be self-administered, which assess the symptoms experienced in the previous two weeks; all items receive a response using a four-point Likert scale (0 = Not at all to 3 = almost every day); 2) An item that extends over the year preceding the award; 3) An item on the effect of his suffering and on his daily performance; 4) An item that extends over one month before the handover; and 5) An item that assesses the presence of suicide attempts in the past. PHQ-A measures functional impairment, suicidal ideation and suicide attempts [24]. To calculate the total of the scale, it is a question of adding all the scores of the items together. The total score can range from 0 to 27 (0 to 4: no severe depression/5 to 9: mild depression/ 10 to 14: moderate/ 15 to 19: moderately severe/ 20 to 27: Severe) [26].

### Statistical analysis

SPSS software v.23 was used to analyze the data. The normality of distribution of the anxiety and depression scores was confirmed since the skewness and kurtosis values fell within the − 2 and + 2 range. Therefore, the Student t test was used to compare two means, whereas the ANOVA test was used to compare three or more means. Correlations between two continuous variables were tested using the Pearson test. Two linear regressions were conducted, taking the anxiety and depression scores as dependent variables.

Regarding suicidal ideation, the Chi-square test was used to compare two categorical variables, whereas the Student t test was used to compare two means. A logistic regression was conducted, taking the presence/absence of suicidal ideation in the last month as the dependent variable. All variables that showed a p < 0.25 in the bivariate analysis were entered as independent ones in the multivariable models. Significance was set at p < 0.05.

## Results

The internal reliability of the scales was as follows: total cyberbullying scale (0.892), sexual cyberbullying in cyberspace (0.824), embarrassing and inserting malicious content in cyberspace (0.833), depression (0.875), and anxiety (0.899).

A total of 520 students enrolled in this study. The mean age was 14.05 ± 0.89 years, with 57.7% females. Other details about the sample can be found in Table 2.

**Table 2:** Sociodemographic and other characteristics of the participants (N = 520)

Variable	N (%)
**Gender**	
Male	220 (42.3%)
Female	300 (57.7%)
**Parents status**	
Married	492 (94.6%)
Separated	28 (5.4%)
**Influence of problems on work**	
Not hard at all	282 (54.2%)
Kind of hard	193 (37.1%)
Very/extremely hard	45 (8.7%)
**Suicidal ideation last month**	
No	451 (86.7%)
Yes	69 (13.3%)
**Sexual cyberbullying in cyberspace**	
Absent	461 (88.7%)
Present	59 (11.3%)
**Embarrassing and inserting malicious content in cyberspace**	
Absent	436 (83.8%)
Present	84 (16.2%)
	**Mean ± SD**
**Age (in years)**	14.05 ± 0.89
**Household crowding index**	1.22 ± 0.56
**Anxiety**	11.85 ± 8.12
**Depression**	6.33 ± 6.10

### Bivariate analysis of factors associated with anxiety and depression

Female gender was significantly associated with higher anxiety and mild depression. Having very/extremely hard influence of problems on daily work, sexual cyberbullying in cyberspace, embarrassing and inserting malicious content in cyberspace were associated with higher anxiety and moderate depression. Also, older age was significantly correlated with more anxiety and depression (Table 3).

**Table 3:** Bivariate analysis of factors associated with anxiety and depression

	Anxiety (mean ± SD)	*p*	Depression (mean ± SD)	*p*
**Gender**		< 0.001		< 0.001
Male	9.65 ± 7.05		5.05 ± 5.66	
Female	13.47 ± 8.48		7.27 ± 6.24	
**Parents status**		0.109		0.082
Married	11.72 ± 8.05		6.22 ± 6.05	
Separated	14.25 ± 9.12		8.29 ± 6.70	
**Influence of problems on daily work**		**< 0.001**		**< 0.001**
Not hard at all	7.75 ± 5.51		3.58 ± 4.27	
Kind of hard	15.51 ± 7.16		8.63 ± 5.51	
Very/extremely hard	21.91 ± 9.51		13.73 ± 7.85	
**Sexual cyberbullying in cyberspace**		**< 0.001**		**< 0.001**
No	11.06 ± 7.70		5.71 ± 5.64	
Yes	18.07 ± 8.71		11.17 ± 7.34	
**Embarrassing and inserting malicious content in cyberspace**		**< 0.001**		**< 0.001**
No	11.00 ± 7.77		5.65 ± 5.69	
Yes	16.26 ± 8.52		9.89 ± 6.88	
**Age**	r = 0.14	**0.001**	r = 0.18	**< 0.001**
**Household crowding index**	r = 0.01	0.907	r= -0.04	0.393

Numbers in bold indicate significant p-values

### Multivariable analysis of factors associated with anxiety and depression

The results of the linear regressions taking anxiety and depression as dependent variables, showed that female gender, having kind of hard and very/extremely hard influence of problems on daily work, sexual cyberbullying in cyberspace, embarrassing and inserting malicious content in cyberspace and older age were significantly associated with more anxiety and depression (Table 4).

**Table 4:** Multivariable analyses

Model 1: Linear regression (using the ENTER method) taking the anxiety score as the dependent variable.				
	Unstandardized Beta	Standardized Beta	*p*	95% CI
Sex (females vs. males*)	3.00	0.18	**< 0.001**	1.88–4.13
Influence of problems on work (kind of hard vs. not at all*)	6.74	0.40	**< 0.001**	5.55–7.92
Influence of problems on work (very/extremely hard vs. not at all*)	12.26	0.43	**< 0.001**	10.18–14.34
Sexual cyberbullying in cyberspace (yes vs. no*)	2.89	0.11	**0.004**	0.93–4.85
Embarrassing and inserting malicious content in cyberspace (yes vs. no*)	2.66	0.12	**0.002**	1.01–4.31
Age	0.78	0.09	**0.014**	0.16–1.40
Parents status (divorced vs. married*)	-0.55	-0.02	0.668	-3.04-1.95
**Model 2: Linear regression (using the ENTER method) taking the depression score as the dependent variable.**				
	**Unstandardized Beta**	**Standardized Beta**	***p***	**95% CI**
Sex (females vs. males*)	1.82	0.15	**< 0.001**	0.94–2.70
Influence of problems on work (kind of hard vs. not at all*)	4.23	0.34	**< 0.001**	3.30–5.16
Influence of problems on work (very/extremely hard vs. not at all*)	8.55	0.40	**< 0.001**	6.92–10.18
Sexual cyberbullying in cyberspace (yes vs. no*)	2.37	0.12	**0.003**	0.83–3.91
Embarrassing and inserting malicious content in cyberspace (yes vs. no*)	2.32	0.14	**< 0.001**	1.03–3.62
Age	0.88	0.13	**< 0.001**	0.39–1.36
Parents status (divorced vs. married*)	0.02	0.001	0.983	-1.94- 1.98

*Reference group; numbers in bold indicate significant p-values. Nagelkerke R^2^ = 0.412 for model 1 and 0.361 for model 2

### Bivariate analysis of factors associated with suicidal ideation

A higher percentage of females compared to males, of those who have very/extremely hard influence of problems on daily work, sexual cyberbullying in cyberspace, and embarrassing and inserting malicious content in cyberspace was significantly found in adolescents who had suicidal ideation in the last month. Moreover, higher mean age, household crowding index, anxiety and moderate depression were significantly found in adolescents who had suicidal ideation in the last month (Table 5).

**Table 5:** Bivariate analysis of factors associated with the presence/absence of suicidal ideation in the last month as the dependent variable

	Absence of suicidal ideation	Presence of suicidal ideation	*p*
**Gender**			0.001
Male	203 (92.3%)	17 (7.7%)	
Female	248 (82.7%)	52 (17.3%)	
**Parents status**			0.245
Married	429 (87.2%)	63 (12.8%)	
Separated	22 (78.6%)	6 (21.4%)	
**Influence of problems on work**			**< 0.001**
Not hard at all	272 (96.5%)	10 (3.5%)	
Kind of hard	153 (79.3%)	40 (20.7%)	
Very/extremely hard	26 (57.8%)	19 (42.2%)	
**Sexual cyberbullying in cyberspace**			**0.012**
No	406 (88.1%)	55 (11.9%)	
Yes	45 (76.3%)	14 (23.7%)	
**Embarrassing and inserting malicious content in cyberspace**			**0.001**
No	388 (89.0%)	48 (11.0%)	
Yes	63 (75.0%)	21 (25.0%)	
**Age**	14.02 ± 0.88	14.25 ± 0.94	**0.046**
**Household crowding index**	1.20 ± 0.54	1.34 ± 0.67	**0.046**
**Anxiety**	10.32 ± 7.10	21.87 ± 7.23	**< 0.001**
**Depression**	5.20 ± 5.16	13.71 ± 6.66	**< 0.001**

Numbers in bold indicate significant p-values

### Multivariable analysis of factors associated with suicidal ideation

Having kind of hard influence of problems on daily work compared to not at all (aOR = 2.42), higher anxiety (aOR = 1.10), higher depression (aOR = 1.13) and higher household crowding index (aOR = 1.86) were significantly associated with higher odds of having suicidal ideation in the last month (Table 6).

**Table 6:** Multivariable analysis: Logistic regression using the ENTER model taking the presence/absence of suicidal ideation in the last month as the dependent variable

Variable	aOR	95% CI	*p*
Sex (females vs. males*)	1.25	0.60–2.59	0.555
Influence of problems on daily work			
Kind of hard vs. Not hard at all*	2.42	1.03–5.65	**0.042**
Very/extremely hard vs. Not hard at all*	2.45	0.77–7.79	0.130
Age	1.02	0.72–1.45	0.923
Parents status (divorced vs. married*)	2.34	0.71–7.75	0.164
Sexual cyberbullying in cyberspace (yes vs. no*)	0.47	0.17–1.30	0.144
Embarrassing and inserting malicious content in cyberspace (yes vs. no*)	1.75	0.76–4.04	0.190
Anxiety	1.10	1.04–1.16	**0.001**
Depression	1.13	1.05–1.21	**0.001**
Household crowding index	1.86	1.15–3.02	**0.012**

*Reference group; numbers in bold indicate significant p-values. Nagelkerke R^2^ = 0.437

## Discussion

### Cyberbullying perpetration, anxiety and depression

Our study showed that sexual cyberbullying perpetrators, and those inserting malicious and embarrassing content in cyberspace have more anxious and depressive symptoms, corroborating the findings of previous studies [9, 27]. The majority of studies in the literature highlighted the effect of bullying victimization and ignored the idea that involvement in sexual bullying has mental health consequences, regardless of involvement as a victim or a perpetrator [28]. Findings suggested that 10% of students in grades 7 to 12 report cyber sexual harassment perpetration [29]. Authors explained that this might be due to increased impulsivity, low empathy, decreased self-esteem, anger, traditional beliefs about masculinity, exposure to pornography, and early sexual initiation [30]. When inserting malicious and embarrassing content and performing cyberbullying, the perpetrator devaluates their victims and therefore, their own worth and self-respect, which increases their anxiety and depression [31]. Additionally, for cyberbullies, the need to harm others originates from lack of empathy [32] indicating lack of social skills [33] and therefore more anxiety and depression [34, 35].

### Suicidal ideation

Higher anxiety and depression were shown significantly associated with more suicidal ideation worldwide [36–39] and specifically among Lebanese [40, 41]. Studies revealed that depressed adolescents experience less pleasure when positively stimulated, but more pleasure in negative experiences; those with suicidal ideation are more prone to appraising positive experiences as negative ones. Also, among adolescents with depressive symptoms the amygdala–hippocampus/brain stem and amygdala–precuneus functions are impaired causing the persistence of depressive symptoms [42] and suicidal ideation among adolescents [43]. Regarding anxiety, worry represents a key indicator of anxiety, and is clinically present among those with suicidal ideation [44]. Despite the various studies showing that anxiety is correlated with suicidal ideation, this association is still inconclusive since anxiety may trigger depressive symptoms associated with suicide [45, 46].

### Socio-demographics and mental health

In accordance with previous studies [47, 48], female gender was associated with more anxiety and depression in our study. Researchers explained that this association is due to the onset of ovarian cycling, during adolescence, contributing to increased anxiety and depressive disorders among females. These reproductive events lead to changes in hormones and hormonal metabolites, and therefore affect brain systems and mood regulation [49]. Additionally, our study was conducted during the COVID-19 pandemic, where the vulnerability to anxiety and depression increases among females as compared to males as shown in previous studies, due to the lack of emotional support [50–53].

Additionally, in line with previous studies conducted in Middle Eastern countries [48, 54], older age was associated with more anxiety and depression in our study. According to researchers, many neurodevelopmental processes that can take place between adolescence and early adulthood, are thought to be correlated with higher levels of anxiety and depression. Modifications to the grey and white matter, such as axonal myelination and increased axon diameter, are examples of neuroplasticity mechanisms allowing environmental experiences to have an impact on brain development, therefore, adolescents became very susceptible to stressful or negative experiences and consequently poorer mental health [55]. Moreover, adolescence and early adulthood are times when persons gain independence, take on responsibilities and often rebel against them, which makes them more susceptible to anxiety and depressive symptoms [56].

Moreover, our findings revealed that higher household crowding index is significantly associated with higher odds of having suicidal ideation, in accordance with previous studies [24, 57, 58]. Researchers raised the question whether household crowding index can directly lead to psychological distress or factors related to housing conditions such as poverty and associated conditions can affect psychological distress. Studies explained that adolescents living in an overcrowded house can develop more mental health illness until their adulthood [59], since they might find it very difficult to escape negative interactions. In addition, the presence of different generations in the same house can increase conflicts or violence [58].

### Influence of problems on daily work, anxiety, depression and suicidal ideation

A significant association was found between having kind of hard and very/extremely hard influence of problems on daily work and anxiety and depression in our study. Researchers explained that the probability of repeating a year, dropping out of school or taking special education increases among adolescents with depression [60, 61]. They justified this correlation by reduced self-worth caused by attention reduction, inability to organize work and memory impairment due to depressive and anxious symptoms [62]. Additionally, connectedness to school (i.e. feeling safe, happy, having friends, etc.) reduces the risk of mental health problems, whereas, low school connectedness lead to higher depression, anxiety and bullying [63].

Last but not least, our findings revealed a significant association between hard influence of problems on daily work and suicidal ideation among adolescents. Students who are affected perceive dropping out as a personal failure, and having to repeat a year as a sort of punishment, causing additional stress and consequently more suicidal ideation [64]. Researchers also revealed that students failing coursework and struggling with reading and writing are also at a higher risk of suicidal ideation [64, 65]. Recent studies conducted in Japan [66] and France [67] showed that school closure due to COVID-19 pandemic decreased psychological burdens among adolescents and reduced suicide rates. Authors explained that lockdown disrupt school harassment and social withdrawal and lead to higher feelings of connectedness and belonging, thus, less suicidal ideation [67, 68].

### Limitations and strengths

Many limitations can be found in our study. First, the cross-sectional type of the study limiting us from establishing causal relationships between variables. In addition, data obtained was self-reported; responders may have over- or under-estimated some questions, leading to information bias. Other factors related to bullying perpetration have not been assessed in the questionnaire, predisposing us to a confounding bias. The cyber bully/ cyber victim scale used in this paper has not been validated in Lebanon yet. A selection bias is present because of the snowball technique followed to collect the data and the inability to know the refusal rate of participation. Despite these limitations, this study was the first in Lebanon aiming to assess the association between cyberbullying perpetration, and anxiety, depression and suicidal ideation among Lebanese adolescents. Although our results consolidate the findings from international studies, our findings cannot be representative of the whole Lebanese population since our sample was recruited in a convenient way.

### Clinical implications

Cyberbullying perpetration and its associated factors reported in this study are significant enough to call for early detection and the creation of prevention strategies for Lebanese adolescents. Females reported higher rates of depression and anxiety than males in our study, which might reflect the need to directly target females with preventative programs developed to be relevant to them. At the school level, effective programs implemented in the school years are needed, aiming to develop social/emotional control, and conflict resolution skills as they might decrease engagement in cyberbullying perpetration among adolescents. Moreover, cyberbullying perpetration in this age group may be avoided by educating families on how to provide appropriate supervision and clear rules and checkup attentively on their kids’ behavior in order to prevent future cyberbullying perpetration.

## Conclusion

Our study shed the light on the association between gender, age, hard daily work problems and sexual cyberbullying on adolescents’ mental health. Our findings also highlight that the association between hard problems affecting daily work, higher anxiety, higher depression, higher household crowding index and higher odds of having suicidal ideation. Future studies should try to understand which personality traits, environmental and social variables might predict the engagement of adolescents in cyberbullying perpetration.

## Data Availability

All data generated or analyzed during this study are not publicly available to maintain the privacy of the individuals’ identities. The dataset supporting the conclusions is available upon request to the corresponding author.
